# Prevalence of Hyperacusis in the General and Special Populations: A Scoping Review

**DOI:** 10.3389/fneur.2021.706555

**Published:** 2021-09-03

**Authors:** Jing Ren, Tao Xu, Tao Xiang, Jun-mei Pu, Lu Liu, Yan Xiao, Dan Lai

**Affiliations:** Department of Otolaryngology Head and Neck Surgery, The Affiliated Hospital of Southwest Medical University, Luzhou, China

**Keywords:** prevalence, hyperacusis, special occupation population, general population, special diseases

## Abstract

**Objectives:** To study the prevalence of hyperacusis in the general population and the special population, and to determine the effect of population differences on hyperacusis.

**Methods:** The two authors followed a scoping review methodology and screened nearly 30 years of English literature in Pubmed, Web of Science, OVID, and EBSCO. Then, the extracted results of each study were discussed in groups and subgroups.

**Results:** The authors selected 42 pieces of scientific literature that met the requirements, studying a total of 34,796 subjects, including the general population (28,425 subjects), the special occupation population (2,746 subjects), and the patients with concomitant diseases (5,093 subjects). The prevalence was 0.2–17.2% in the general population, 3.8–67% in the special occupation population, and 4.7–95% in the patients with special diseases. It was found that in the general population, the high prevalence occurs in adolescents and older adults. The prevalence of hyperacusis in women is significantly higher than in men. In people with hearing disorders, the prevalence of hyperacusis is significantly higher than in people with normal hearing. Various diseases (such as Williams syndrome, tinnitus, and autism), as well as various occupations (musicians, music students, teachers, and others), have been found to be high risk factors for hyperacusis.

**Conclusion:** The high prevalence of hyperacusis and the large differences between reported prevalence in different studies deserves our great attention. Additionally, in order to increase the comparability of the studies, a standardized set of criteria are needed to study the prevalence of hyperacusis.

## Introduction

Hyperacusis is defined as a reduced tolerance to sounds of average intensity, sometimes accompanied by painful sensitivity to ordinary environmental sounds, with perceptual, psychological, and social dimensions (1). The sounds may be perceived as uncomfortably loud, unpleasant, frightening, or painful (2). When patients with hyperacusis experience pain at much lower sound levels than listeners with normal hearing, then it can be described as pain hyperacusis. And when moderately intense sounds are judged to be very loud compared with what a person with normal hearing would perceive, it can be called loudness hyperacusis. Additionally, hyperacusis also includes annoyance hyperacusis and fear hyperacusis (3).

Hyperacusis patients acutely capture small sounds or a particular sound, including screams, whistles, thunder, and rattling of dishes, as well as less obtrusive noises, such as televisions, telephones, and cars, then consciously focus on these sounds that make up the background of daily life, thus diverting attention to sounds that should be ignored (4). They often wear earplugs to avoid this type of sound stimulation. In addition, hyperacusis appears to be associated with both tinnitus and hearing impairment (5). Symptoms of hyperacusis include disturbed sleep, fatigue, negative emotional well-being, anxiety, and concentration difficulties (6). Klein's research has observed that children with Williams syndrome (WS) often exhibit behavioral responses to offensive sounds associated with hyperacusis, such as covering ears with hands, crying, and cringing (7).

Many unknowns about hyperacusis remain unexplored and there are currently no formal clinical guidelines for hyperacusis (8). Perhaps it is why more and more literature studying and exploring current situation of hyperacusis. Not limited to the general population, the exploration of the hyperacusis in special population is also gradually deepened. Although there has been a growing body of studies on hyperacusis in recent years, the consistency in methods and studied populations across these studies is limited, which can make comparisons between studies challenging. This is well-presented in studies of its prevalence. Although prevalence studies in different populations are helpful in identifying the epidemiological characteristics of hypreacusis, current studies are chaotic. Different studies have mentioned different prevalence in different populations (8–11). A recent systematic review considering hyperacusis in the childhood and adolescence concluded that making comparisons was not possible at present (12). While it is also not possible to generalize across studies, some data are available (13). Differences in age, sex, occupation, and comorbidities among studies make comparison difficult. However, group comparisons and even subgroup comparisons can increase the credibility of reviews.

The purposes of this review are as follows:

◦To compare the prevalence of hyperacusis across populations of differing age, gender, concomitant diseases, hearing disorders, and specific occupations, and to assess the impact of these differences on the prevalence of hyperacusis.

◦To collect studies on hyperacusis, defining characteristics of hyperacusis patients, as well as cataloging the characteristics of these studies.

## Methods

Due to the broad and exploratory nature of the research questions, a scoping review based on the methodological framework proposed by Arksey and O'Malley (14) is most suitable methodology, which is carried out in five stages: (1) identifying the research question; (2) identifying relevant studies; (3) study selection; (4) charting the data; (5) collating, summarizing and reporting the results.

### Search Strategy and Data Sources

Two reviewers limited the search period from 1990 to 2020 (The last literature search was conducted on January 17, 2021), and screened the literature separately, with one reviewer searching Pubmed and Web of Science, while the other screened the literature in OVID and EBSCO. Afterwards, two reviewers searched Baidu, Google, and other non-academic websites, and included the studies that meet the criterion in this study. Given the lack of a clear definition of hyperacusis and considering that some authors may compare misophonia and phonophobia with hyperacusis, the first search category is as follows: hyperacusis OR misophonia OR phonophobia OR noise sensitivity. The meaning of the five words, epidemiology, prevalence, morbidity, occurrence and incidence, is similar, so the second category is as follows: epidemiology OR prevalence OR morbidity OR occurrence OR incidence. By combining the first category and the second category, the retrieval results were obtained.

### Study Selection

Two authors screened the titles, abstracts and (or) the full text according to the research question and PICOS protocol (the Population, Intervention, Comparisons, Outcomes and Study design), and then extracted and recorded the selected literature's relevant data into the table established before final selecting. The criteria for PICOS are as follows:

Population, general population and special population with hyperacusis of all ages, including patients with hyperacusis of special diseases and (or) special professionals;

Intervention, none;

Comparisons, the control can be or cannot be set according to the needs of the research plan;

Outcomes, prevalence, or consequences related to hyperacusis;

Study design, all study designs, but case series and case studies were not included.

In addition, another inclusion criterion was also used by the two authors: the included articles must contain the prevalence of hyperacusis, or the prevalence of hyperacusis can be calculated. If the definition of hyperacusis is mentioned, a questionnaire is used to assess hyperacusis, or a loudness discomfort area is measured, then the study will be given priority for inclusion.

### Data Extraction

Before data extraction, a data extraction table is formulated in excel and piloted on two included records. Then it is modified following group discussions. Data items for charting included: year and country of publication, study design (e.g., control study, retrospective study, prospective study), setting (e.g., special occupation, children, patients with special diseases), basic information of subject (number, age, gender, disease), method to evaluate the hearing status, definition of hyperacusis, diagnosis way, data collection for hyperacusis (the way to collect data), conclusion, prevalence of hyperacusis (mentioned in the literature or self-calculated), 95% CIs, factor linked to prevalence (prevalence of hyperacusis in people with normal hearing, hearing loss, or tinnitus), prevalence on controls, and other outcomes (other important prevalence or outcomes).

## Results

### Study Selection

**Figure 1:**
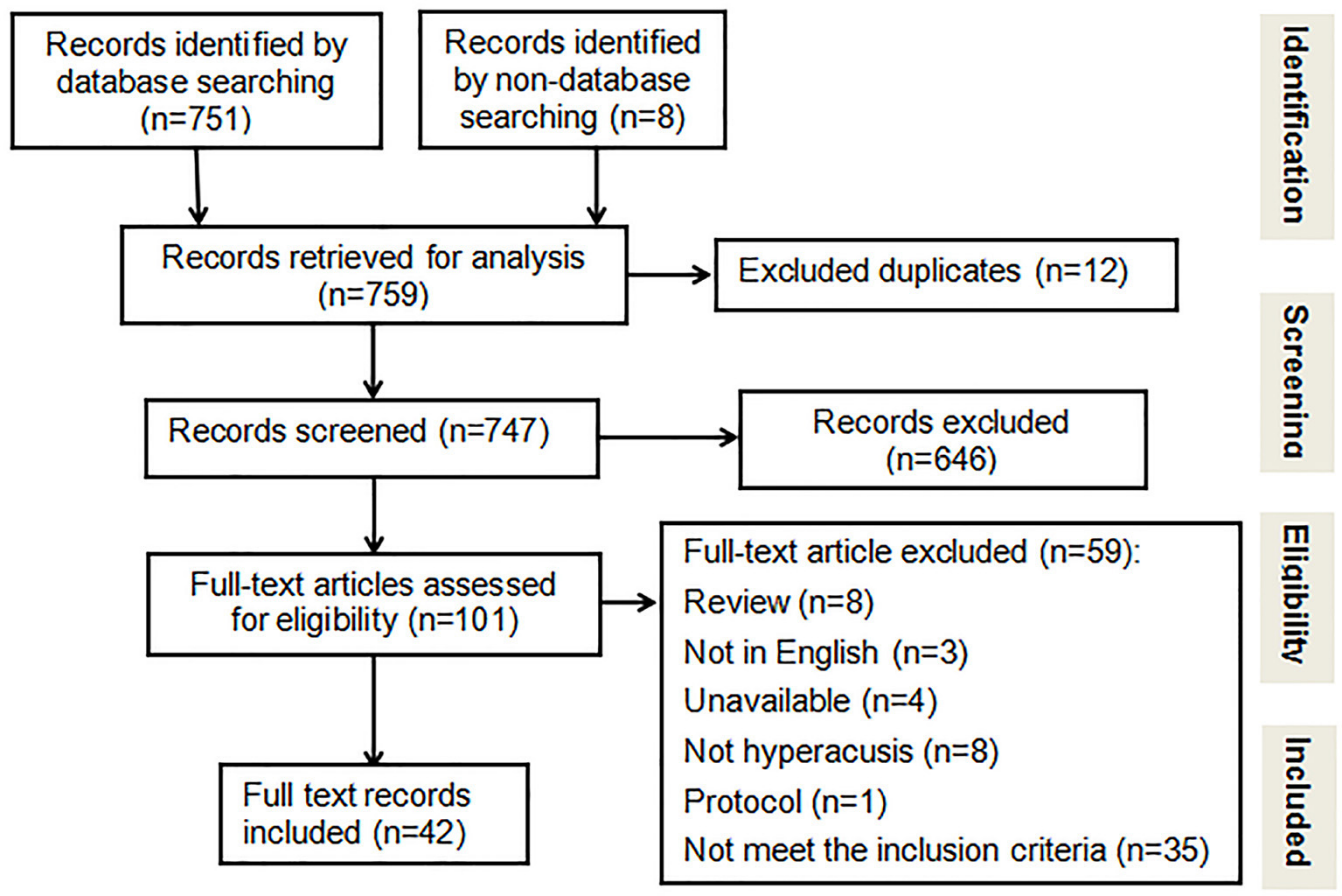
PRISMA flow diagram of the study selection process.

As shown in Figure 1, by combining the first category and the second category, as well as non-database retrieval results, 759 retrieval results are obtained. After removing 12 duplicates and excluding 646 studies by title or (and) abstract, 101 articles met the requirements. Subsequently, studies were screened out for the reasons shown in Figure 1, yielding the final count of 42 articles that were included in this review. Among the 42 included studies, 40 were prevalence studies. Two of the studies described the proportion of patients using the term “incidence” (15, 16), but reviewed by two reviewers and a temporary reviewer, the original studies did not indicate that the patients were new cases. Therefore, we decided to add these 2 studies to our review as prevalence studies.

### Study Characteristics

**Figure 2:**
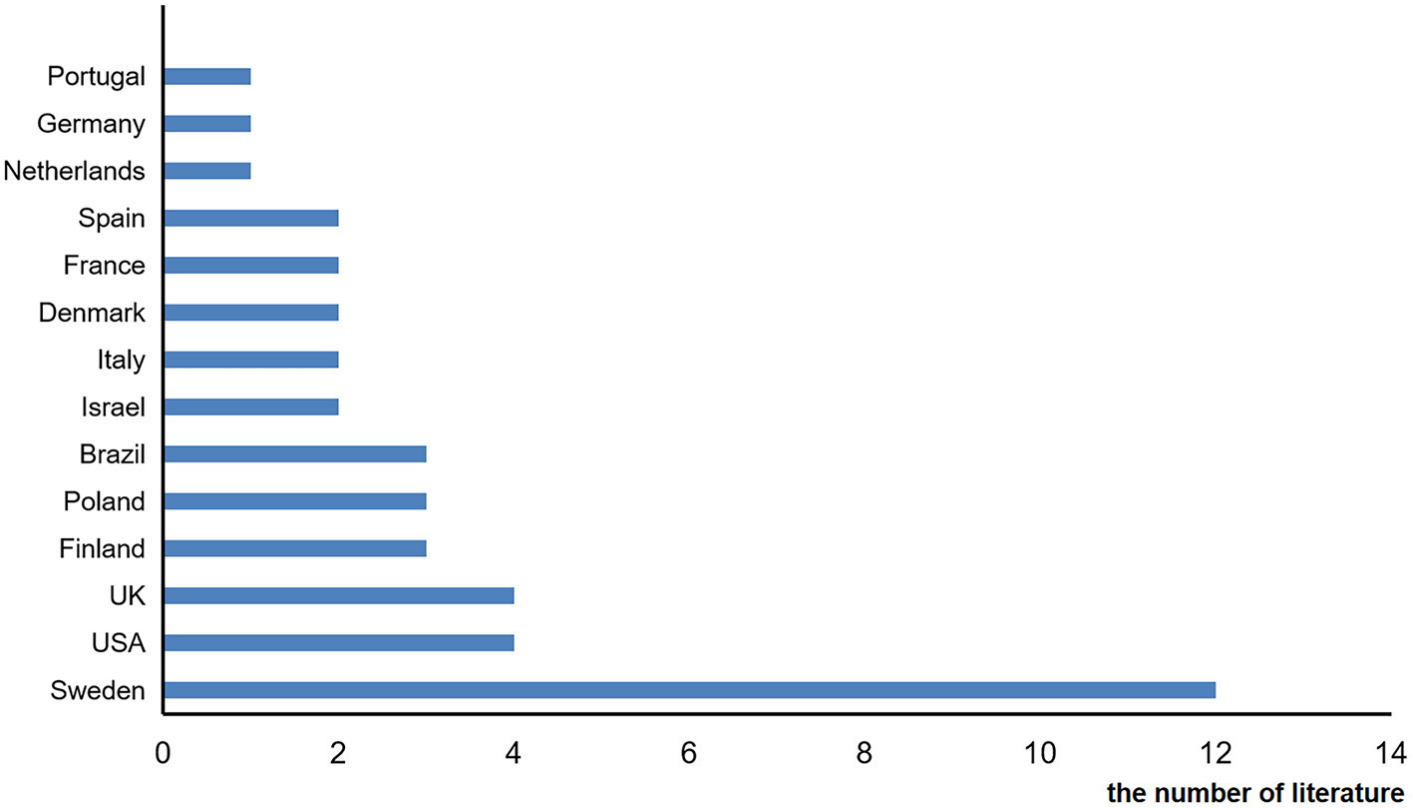
Areas covered by the included literature.

The included studies came from different countries: Sweden, USA, Finland, UK, Poland, France, Brazil, Spain, Netherlands, Germany, Portugal, Israel, Italy, and Denmark (Figure 2).

Information from 42 articles was extracted and recorded. As shown in Table 1, they were published since 1990. The number of individuals in the studies range from 18 to 12,000. And they cover a wide range of ages, from 1 to 98.

**Table 1:** Characteristics of all included studies.

**References**	**Country**	**Study design**	**Setting**	**Sample**					**Definition or Diagnosis based on**	**Data collection for hyperacusis**	**Conclusion**
				**Number of participants**	**Age**	**Male:Female**	**With special diseases**	**Method to evaluate the hearing status**			
Klein et al. (7)	USA	Controlled study	Pediatric patients	130	1–28 y	68:62	Williams syndrome	Not provided	Based on the question “Has your child ever been unusually frightened by certain sounds?”	Questionnaire to parents	Prevalence for hyperacusis in patients with Williams syndrome was significantly higher than in the general population
Axelsson et al. (17)	Sweden	A follow-up Study	Musicians	53	Mean age: 41.2 y	Not provided	No	Tested on pure tone audiometry	Not provided	A detailed questionnaire	It seems surprising that pophock musicians after performing for 26 years have such well-preserved hearing
Rosenhall et al. (9)	Sweden	Prospective controlled study	Pediatric patients	199	Study group:1.2–21.3 y	153:46	Autism	Audiometry/ auditory brainstem response examination	Intolerance to broadband CLICKS at 80 dB HL	Testing for hyperacusis	The study emphasizes the need for auditory evaluation of individuals with autism in order to refer those with pronouced to profound hearing loss for aural habilitation and to follow those with mild to moderate hearing loss because of the risk of deterioration
Skarzyn'ski et al. (18)	Poland	Epidemiological studies/survey	General poplation	12,000	Not provided	Not provided	No	A questionnaire	Not provided	Not provided	The data obtained during our epidemiological survey indicate a need to change the way physicians, organizers of healthcare and those who bear the costs of treatment think about patients with tinnitus
Andersson et al. (19)	Sweden	Cross-Sectional	General population	1,157	16–79 y	539:608	No	Questionnaire	Response of 'yes' to the question regarding sensitivity to every sounds	Questionnaire	Hyperacusis is a common problem
Herraiz et al. (20)	Spain	Transversal descriptive	Patients	213	20–98 y	83:130	Tinnitus	Audiological method	Intolerance to sound threashold level < 90 dB	LDL and questionnaire	A physiopathological relation between tinnitus and hyperacusis could be explained by the high prevalence of both symptoms in the same population
Kähärit et al. (21)	Sweden	Descriptive and cross-sectional study	Musicians	139	26–47 y	96:43	No	Pure-Tone audiometry	Defined as hypersensitivity to the loudness of sounds, including a decreased pure tone, and HLL of specific sounds normally	Questionnaire	It is important to evaluate all kinds of hearing problems (other than hearing loss) in musicians
Khalfa et al. (22)	France	Controlled study	Pediatric patients	22	9–17 y	18:4	Autism	Audiological method	LDL lower than 80 dB HL	LDL	Smaller auditory dynamic ranges were found in the autistic group than in the control group, as well as increased perception of loudness, indicating hyperacusis in subjects with autism
Olsen Widén and Erlandsson (23)	Sweden	Cross-Sectional design	Adolescents	1,285	13–19 y	620:665	No	Questionnaire	Self reported based on the question“Do you consider yourself to be oversensitive to noise?” “Have you ever experienced pain in the ears associated with loud noise?”	Questionnaire	Age-related differences in the prevalence rates of experienced tinnitus and noise sensitivity were found to be significant. Older students reported such symptoms to a greater extent than younger students did
Liriano et al. (24)	Brazil	Clinical prospective	Patients	18	18–60 y	7:11	With Bell's palsy	Audiological method	Defined as hypersensitivity to everyday common sounds perceived by patients as unbearable, strong and painful.	LDL	The frequency of complaints of hyperacusis in-patients with Bell's palsy is similar to that of the general population
Levitin et al. (15)	USA	Clinical prospective	Patients	118	10-30 y	61:57	With Williams syndrome	Audiological method	Lowered hearing thresholds	Audiological methodand questionnaire	The results confirm anecdotal reports of an unusual auditory phenotype in WS
Gothelf et al. (25)	Israel	Cross-Sectional design	Patients	49	1–35 y	20:29	With Williams syndrome	The questionnaire and Audiologic testing	Based on the question “Has your child ever been unusually frightened by certain sounds?”and “Has your child ever been bothered by sounds in the past?”	Hyperacusis Screening Questionnaire and Audiologic testing	Hyperacusis in Williams syndrome (WS) is associated with a high-frequency hearing loss resembling the configuration of noise-induced hearing loss
Blomberg et al. (26)	Sweden	Cross-Sectional design	Patients	38	10–50 y	25:13	With Williams syndrome	Questionnaire	Discription of hyperacusis	Hyperacusis Questionnaire (HQ)	This report supports a hypothesis that fears and anxiety could be associated with hyperacusis in the WS population
Coelho et al. (27)	USA	Cross-Sectional design	School-aged children	506	5–12 y	263:236	No	Hearing tests	Criteria: bothered and annoyed by sounds and LDLs in the 5th percentile at least in one frequency at least in one ear	Questionnaires, interviews and estimates of LDL	Hyperacusis in children is prevalent, and should be considered in clinical examinations
de Klaver et al. (28)	Netherlands	Clinical prospective	Patients	40	Mean age: 41.9 y	2:38	Complex regional pain syndrome related dystonia	Pure-tone audiogram thresholds (PTT), speech reception thresholds (SRT)	A UCL threshold below 100 dB indicate the presence of hyperacusis	Estimates of UCL	Hyperacusis is common among severely affected patients with CRPS related dystonia
Laitinen and Poulsen (29)	Finland	Clinical prospective	Musicians	145	Not provided	88:57	No	Self reported	Hyperacusis was defined as “abnormal sensitivity to everyday sound levels or noises. Often there is also sensitivity to high pitched sounds”	Questionnaire	Education is needed to change musicians' opinion of hearing conservation and hearing protectors
Hasson et al. (30)	Sweden	A epidemiological study	Musicians	250	23–68 y	155:93	No	Self reported	Not provided	Questionnaire	The results indicate that self-reported hearing problems are associated with perceived poorer psychosocial environment, as well as mental health symptoms and stress
Hannula et al. (5)	Finland	Cross-Sectional, population-based, and unscreened	Older Adults	850	54–66 y	383:467	No	Otological examination, pure tone audiometry, questionnaire survey	Defined as particularly sensitive to loud sounds	Questionnaire (questions were used to screen self-reported hearing problems.)	The results indicate that self-reported hearing difficulties are more frequent than hearing impairment defined by audiometric measurement
Toppila et al. (31)	Finland	Prospective study	Musicians	63	22–52 y	38:25	No	Audiometer measurements	Not provided	A questionnaire items with a 5-point Likert scale of never, seldom, sometimes, often, always	The musicians' hearing loss distribution corresponded to that of the general population, but highly exposed musicians had greater hearing loss at frequencies over 3 kHz than less-exposed ones
Baguley et al. (32)	Spain	Retrospective case review	Childhood and adolescence	88	<18 y Mean age: 13.8 y	44:44	Complaint of tinnitus	Audiometric test	Not provided	Structured questionnaire	Epidemiological data for childhood tinnitus reported previously should be interpreted with caution
Hebert et al. (33)	France	Control study	Patients	63	Mean age: 54 y	38:25	Tinnitus	Audiological method	Auditory sensitivity scores increase	Hyperacusis questionnaire and discomfort thresholds	Our results show that auditory sensitivity is enhanced in tinnitus subjects compared with non-tinnitus subjects, including subjects with normal audiograms
Landalv et al. (34)	Sweden	Cross-Sectional	Adolescents	242	15–19 y	132:108	No	A questionnaire	Based the question “Do you experience yourself being overly sensitive to sound?”	The questionnaire included self-perceived auditory symptoms.	Health promotive strategies should focus on changing not merely individual attitudes, but also societal norms and regulations in order to decrease noise induced auditory. symptoms among adolescents
Guimarães et al. (35)	Brazil	Retrospective study	Patients	309	Mean age: 53 y	169: 140	Complaint of tinnitus	Audiological evaluation	Defined as hypersensitivity to sound, in which a common sound stimulus is perceived as extremely intense or uncomfortable.	Questionnaire	The degree of annoyance due to tinnitus had no correlation with the presence of hyperacusis
Danesh et al. (36)	USA	Clinical prospective	Patients	55	4–42 y	46:9	Asperger's Syndrome (AS)	A home-developed case-history survey	Defined as being sensitive to the loudness of sounds which are not considered loud by others	Hyperacusis Questionnaire (HQ)	Hyperacusis also appears to be more prevalent in the AS population than in the ASD population at large
Meuer and Hiller (10)	Germany	Clinical prospective	Teachers	1,468	21–69 y	608:860	Self-reported hearing disorders	Online survey (self-reported)	Not provided	Questionnaire of the German Tinnitus League	The frequent prevalence of hearing disorders in German teachers points to a need of better noise prevention in German schools as one priority of occupational safety
Rodrigues et al. (11)	Portugal	Clinical prospective	Music students	240	11–37 y	132:108	No	Self-reported	Not provided	The questionnaire including ear symptoms	These findings reflect the importance of starting intervention in relation to noise risk reduction at an early stage, when musicians are commencing their activity as students
Halevi-Katz et al. (37)	Israel	Prospective study	Musicians	44	20–64 y	36:8	No	Audiometric hearing threshold assessment	Beginning with a yes/no question (e.g., “Have you ever experiencedhyperacusis?”), and rating the extent:Never, seldom, sometimes, often, or always within a certain musical setting, fromone to five.	The questionnaire including ear symptoms	Weekly hours playing were found to have a greater effect on hearing loss in comparison to years playing. Use of hearing protection was not linked to the extent of exposure to amplified music
Luders et al. (38)	Brazil	Prospective study	Musicians	100	18–64 y	70:30	No	The questionnaire	Intolerance to loud sounds	The questionnaire	The presence of auditory symptoms, especially tinnitus, among musicians reinforces the need for implementation of hearing conservation programs for this profession
Hall et al. (4)	UK	A prospective UK population-based study	Children	7,097	11 y	3,485:3,612	No	Audiological method and parental questionnaires	Responded affirmatively to the question “Do you have a hard time tolerating everyday sounds that you believe most other people can tolerate?”	Hyperacusis interview	The prevalence of hyperacusis in the population of 11-year-old UK children is estimated to be 3.7%. It is more common in boys
Paulin et al. (39)	Sweden	A large-scale population-based questionnaire study	General population	3,406	18–79 y	1,508:1,898	No	The questionnaire	Perceived as more annoying or disturbing than normal, resulting in symptoms such as headache, fatigue, and concentration difficulties	11-item Noise Sensitivity Scale (NSS)	High age, female sex, and high education were associated with hyperacusis
Rosing et al. (40)	Denmark	A prospective study and a retrospective case review	Children	69	5–18 y	38:31	Complaint of tinnitus and/or hyperacusis	Audiometry	Experience of reduced tolerance of sound of moderate or lowintensity	Not provided	A majority of children with tinnitus and/or hyperacusis are seen in settings designed for adult audiological rehabilitation
Basjo et al. (41)	Sweden	A cross-sectional study	Children	416	9 y	204:212	No	Audiological method and a questionnaire	Defined as abnormal sensitivity to everyday sound at normal loudness	A questionnaire containing 11 questions, questions 6–9 addressed possible hyperacusis	The prevalence of hyperacusis in the population of 9-year-old Swedish children is low
Pawlaczyk-Luszczyska et al. (16)	Poland	A prospective study	Young People	58	18–28 y	29:29	Portable audio playersusers	Hearingexamination, questionnaire surveys and Self-assessment of hearingcapabilities	Not provided	Self-assessment	Data presented here did not support thethesis that frequent usage of PAPs was associated with higher risk of worsening hearing ability in young adults
Pawlaczyk-Luszczynska et al. (42)	Poland	Control study	Music students	168	18–31 y	86: 82	No	Hearing examinations	Not provided	A questionnaire survey	The results confirm the need for further studies and development of a hearing conservation program for music students
Ralli et al. (43)	Italy	A prospective Study	Children	109	4–7 y	50:59	No	Components of speech and language through the administration of the Italian versions of six tests	Defined as a reduced tolerance to sounds of average intensity	Observation of children's reactions to selected sounds and with the use of a questionnaire	The results suggest some difficulties in lexical access and the use of shorter sentences by children with hypersensitivity to sound
Aazh et al. (2)	UK	A retrospective study	Young patients	62	4–18 y	32:30	Seeking help for tinnitus and/or hyperacusis	Audiometric thresholds and ULLs (across all frequencies from 0.25 to 8 kHz)	Hyperacusis was considered as present if the average ULL at 0.25, 0.5, 1, 2, 4, and 8 kHz for the ear with the lower average ULL, which is denoted as ULLmin, was <77 dB HL	ULL	Among children and adolescents seen at an audiology outpatient clinic for tinnitus and hyperacusis, hyperacusis diagnosed on the basis of ULLs is very prevalent and it is often characterized by lower ULLs at 8 than at 0.25 kHz
Rashid et al. (44)	UK	Retrospective study	Children	80	7–16 y	34:46	FHL	Audiological method	Defined as over sensitivity to loud sounds	Not provided	A significantly larger percentage of children in the control group (auditory processing disorder) had significant hyperacusis compared to children in the FHL group
Paulin et al. (6)	Sweden	A retrospective study	General population	856	18–79 y	339: 517	No	Not provided	Responded affirmatively to the question “Do you have a hard time tolerating everyday sounds that you believe most other people can tolerate?”	Self-reported and the 11-item Noise Sensitivity Scale (NSS)	The results suggest that worrying about aspects at work, perceiving low social support, and not perceiving being rewarded at work are associated with hyperacusi
Ralli et al. (45)	Italy	Control study	Children	30	4–12	25:5	attention deficit hyperactivity disorder	Auditory evaluation	Described as a reduced tolerance to sounds of average intensity, sometimes accompanied by painful sensitivity to ordinary environmental sounds, with perceptual, psychological and social dimensions	a questionnaire to parents (PQ) and an interview with children (CI)	The preliminary results of our study confirm a higher number of children with hyperacusis among those with ADHD compared to that of the general population of a similar age.
Cederroth et al. (46)	Sweden	Control study	Patients	1,984	Mean 47.7	950:1.034	Tinitus	The questionnaire	The reduced tolerance to general everyday sounds is also known as hyperacusis	Based on the question“Over the last week, have external sounds been a problem, being too loud or uncomfortable for you when they seemed normal to others around you?”	The present study suggests that hyperacusis is strongly associated with tinnitus, and that this relationship increases with severity
Couth et al. (47)	UK	Prospective study	Musicians	76	18–26	36:40	No	Audiological method and questionnaires	Defined as “an abnormal sensitivity to everyday sound levels or noises. Often there is also sensitivity to high pitched sounds. In some circumstances, certain sounds may become painfully loud”	The Modified Khalfa Hyperacusis Questionnaire	we did observe a higher prevalence and severity of hyperacusis with higher levels of noise exposure, most of which was from recreational activities
Nemholt et al. (48)	Denmark	A cross-sectional Study	Children	501	10.9–16.6 Mean 13.7	226:275	No	Audiological method	Defines hyperacusis as “abnormal, lowered tolerance to sound.” Hyperacusis is used as a general term for decreased sound tolerance, regardless of the emotional impact or source of sound	A questionnaire	We found a strong association between ST (spontaneous tinnitus) and hyperacusis. hyperacusis was more common in ST

The target populations of the studies are also diverse. Some studies (*n* = 11) aimed at special occupation groups, for example, 10 studies about musicians and music students (11, 17, 21, 29–31, 37, 38, 42, 47), and 1 study about teachers (10). Some studies focus on patients with cognitive impairment (*n* = 7), such as patients with WS (7, 15, 25, 26), autism (9, 22), and Asperger's syndrome (AS) (36). In addition, some studies (*n* = 12) are specific to patients with other comorbidities [tinnitus (2, 20, 32, 33, 35, 40, 46), hearing disorders (HD) (10, 44), Bell 's palsy (24), attention deficit hyperactivity disorder (ADHD) (45) and complex regional pain syndrome related dystonia (CRPS related dystonia) (28)]. Other studies have focused on the general populations (4–6, 16, 18, 19, 23, 27, 34, 39, 41, 43, 48).

#### Definitions of Hyperacusis

In 25 pieces of selected literature, the definition of hyperacusis is mentioned (2, 4, 6, 7, 15, 19, 21, 24–27, 29, 30, 33–36, 39, 40, 43–48) (Table 2). Additionally, all use the word hyperacusis.

**Table 2:** The definition of hyperacusis mentioned in 25 studies.

Klein et al. (7	Consistently exaggerated or inappropriate responses or complaints of uncomfortable loudness to sounds that are neither intrinsically threatening nor uncomfortably loud to a typical person
Andersson et al. (19)	Consistently exaggerated or inappropriate responses or complaints of uncomfortable loudness to sounds that are neither intrinsically threatening nor uncomfortably loud to a typical person (7)
Kähärit et al. (21)	Hypersensitivity to the loudless of sounds, including a decreased pure tone, and uncomfortable loudless level of specific sounds normally not experienced as loud, uncomfortable or annoying
Liriano et al. (24)	Hypersensitivity to common everyday sounds, perceived as unbearable, strong, or painful
Levitin et al. (15)	Lowered hearing thresholds, that is, detectability thresholds for soft sounds
Gothelf et al. (25)	Oversensitivity or excessive perception of normal environmental sounds
Blomberg et al. (26)	An unusual oversensitivity to sound or noise with a high volume or strength or to specific sound or noise (regardless of volume or strength), which are acceptable for most people
Coelho et al. (27)	Lowered loudness discomfort levels (LDL) associated with an abnormal annoyance to sounds
Laitinen and Poulsen (29)	Abnormal sensitivity to everyday sound levels or noises. Often there is also sensitivity to high pitched sounds
Hasson et al. (30)	Pain as a response to loud noises or high sensitivity to surrounding sounds
Hebert et al. (33)	A hypersensitivity to moderate sounds, which can be conceived as a “pathology” of loudness
Landälv et al. (34)	An extreme sensitivity to everyday sounds of low intensity
Guimarães et al. (35)	A manifestation of an increased of central auditorypathways gain and can be considered a pre-tinnitus state
Danesh et al. (36)	Consistently exaggerated or inappropriate responses to sounds that are neither threatening nor uncomfortably loud to a typical person
Hall et al. (4)	An abnormal lowered tolerance to sound
Paulin et al. (39)	A condition in which exposure to everyday sounds is perceived as more annoying or disturbing than normal, resulting in symptoms such as headache, fatigue, and concentration difficulties
Rosing et al. (40)	The experience of reduced tolerance of sound of moderate or low intensity
Ralli et al. (43)	A reduced tolerance to sounds of average intensity, sometimes accompanied by painful sensitivity to ordinary environmental sounds, with perceptual, psychological, and social dimensions
Aazh et al. (2)	Intolerance of everyday sounds that causes significant distress and impairment in social, occupational, recreational and other day-to-day activities
Rashid et al. (44)	Over-sensitivity to loud sounds
Paulin et al. (6)	Characterized by negative reactions to sounds at lower levels than to which the majority reacts
Cederroth et al. (46)	The reduced tolerance to general everyday sounds
Couth et al. (47)	An abnormal sensitivity to everyday sound levels or noises. Often there is also sensitivity to high pitched sounds. In somecircumstances, certain sounds may become painfully loud
Nemholt et al. (48)	Abnormal, lowered tolerance to sound. A general term fordecreased soundtolerance, regardless of the emotional impact or source of sound
Ralli et al. (45)	Described as a reduced tolerance to sounds of average intensity, sometimes accompanied by painful sensitivity to ordinary environmental sounds, with perceptual, psychological and social dimensions

#### Diagnosis and Evaluation

Diagnostic criteria vary from study to study. For example, in two studies that asked questions to diagnose hyperacusis (23, 37), Widen and Erlandsson's (23) diagnosis is based on the question “Do you consider yourself to be over sensitive to noise?” Or “Have you ever experienced pain in the ears associated with loud noise?” Meanwhile, Halevi-Katz (37) further classified responses into five grades: never, seldom, sometimes, often, or always. There were 8 other studies that were diagnosed by asking questions (4, 6, 7, 19, 23, 25, 34, 37). Only two studies used the same questions for diagnosis (4, 6). The discomfort thresholds (LDL, UCL, intolerance sound level, etc) had been measured in 9 studies (2, 9, 20, 22, 24, 25, 27, 28, 33), of which 6 used lowered discomfort loudness as the diagnostic criteria (2, 9, 15, 20, 22, 28). But only 2 presented the same discomfort loudness (80 dB HL) (9, 22). Different definitions were also used to diagnose hyperacusis (5, 21, 24, 26, 29, 33, 35, 36, 38–41, 43–48). Coelho (27) used both the hyperacusis definition and the discomfort loudness to diagnose. Nine studies did not even give their diagnostic criteria (10, 11, 16–18, 30–32, 42).

As for methods to evaluate hearing status, there are many types and combinations. Except for 2 studies that do not provide listening evaluation methods (6, 7), the rest of the studies describe their own listening assessment methods, with 10 studies using different questionnaires (18, 19, 23, 26, 34, 38, 39, 46) or surveys (10, 36), while 21 studies (2, 9, 15, 17, 20–22, 24, 27, 28, 31–33, 35, 37, 40, 42–45, 48) carrying out by a method of audiology, such as pure tone audiometry (17, 21), hearing tests (27, 32, 42), auditory brainstem response examination (9), and other methods. Also, some studies used both (4, 5, 16, 25, 41, 47). Three other studies (11, 29, 30) evaluated hearing using self-reporting.

### Result Assessment and Discovery

The results of each study are shown in Table 3. In the general population, the prevalence of hyperacusis is 0.2% (41)−17.2% (5). In most cases, women have a higher prevalence rate than men. In a study of the prevalence of hyperacusis in the general population, the prevalence increased with age (19). Additionally, in the special occupation groups, the prevalence is 3.8% (17)−67.0% (38). In people with specific conditions (WS, tinnitus, and autism), the prevalence ranges from 4.7% (15) to 95.0% (7). Hyperacusis may be related to certain factors, such as HD and tinnitus, but hyperacusis also exists in the normal hearing population. As described below, different groups of populations were further discussed.

**Table 3:** Outcome assessment.

**Study**	**Prevalence (%)**	**95% CIs**	**Factor linked to prevalence (%)**			**Prevalence on controls (%)**	**Other outcome**
			**Normal hearing**	**Hearing impaired**	**Tinnitus**		
Klein et al. (7)	95.0					12.0	
Axelsson et al. (17)	3.8			11.3	3.8		
Rosenhall et al. (9)	18.0	11.6–26.8				0.0	
Skarzynski et al. (18)	15.0						
Andersson et al. (19)	8.6	7.0-10.0	6.8	19.4			16–30y 6.0% 31–50y 11.0% 51–79y 15.0%
Herráiz et al. (20)	63.0			83.0	63.0		
Kähärit et al. (21)	45.3						Female > Male
Khalfa et al. (22)	63.0					27.0	
Widén and Erlandsson (23)	17.1						
Liriano et al. (24)	5.5						
Levitin et al. (15)	4.7						Odynacusis 79.8%
Gothelf et al. (25)	83.7		30	70			
Blomberg et al. (26)	13.0						Female > Male
Coelho et al. (27)	3.2	2.0–5.2		6.6	8.3		Phonophobia 9% in children
de Klaver et al. (28)	38.0						
Laitinen and Poulsen (29)	7.0						Tinnitus:24%
Hasson et al. (30)	14.0						
Hannula et al. (5)	17.2						Man: 11.5% Women: 21.8%
Toppila et al. (31)	41.0						
Baguley et al. (32)	39.0						Severe tinnitus: 18%
Hebert et al. (33)	60.0					20.0	
Landälv et al. (34)	3.3	1.7–6.4					
Guimarães et al. (35)	18.4						Men: 22.4% Women: 15.3%
Danesh et al. (36)	69.0						Both hyperacusis and tinnitus: 31.0%
Meuer and Hiller (10)	24.9(in all) 67.3(in HD)						Hyperacusis + tinnitus: 21.2% Hyperacusis + hearing loss 13.1%
Rodrigues et al. (11)	30.8						
Halevi-Katz et al. (37)	40.0 in dummers						
Lüders et al. (38)	67.0						
Hall et al. (4)	3.7	3.25–4.14					Male > Female
Paulin et al. (39)	11.1						
Rosing et al. (40)	12.8						Hyperacusis + tinnitus: 15.7%
Båsjö et al. (41)	0.2						Tinnitus: 5.3%
Pawlaczyk-Luszczyska et al. (16)	6.9	2.3–17.0					Tinnitus: 5.2%
Pawlaczyk-Łuszczyńska et al. (42)	36.3	29.4–43.8				Non-music students: 11.9	
Ralli et al. (43)	13.8						
Aazh et al. (2)	58.0						Severe hyperacusis: 17%
Rashid et al. (44)	35.0					Auditory processing disorder: 62.0	
Paulin et akl. (6)	5.5						Female > Male
Ralli et al. (45)	36.7					13.3	Tinnitus in study group:13.3% Tinnitus in control group:6.7%
Cederroth et al. (46)	58.6	3.51			Severe Tinnitus (Self-Reported):78.7 SevereTinnitus(THI ≥ 58):86.6	Non-tinnitus human: 24.4	
Couth et al. (47)	26.3					Non-musicians:19.2	Tinnitus in musician:73.7% Tinnitus in non-musician:68.1%
Nemholt et al. (48)	14.6				8.0		Any tinnitus:66.9%; noise-inducedtinnitus:35.7%; spontaneous tinnitus:53.7%

#### Age

**Figure 3:**
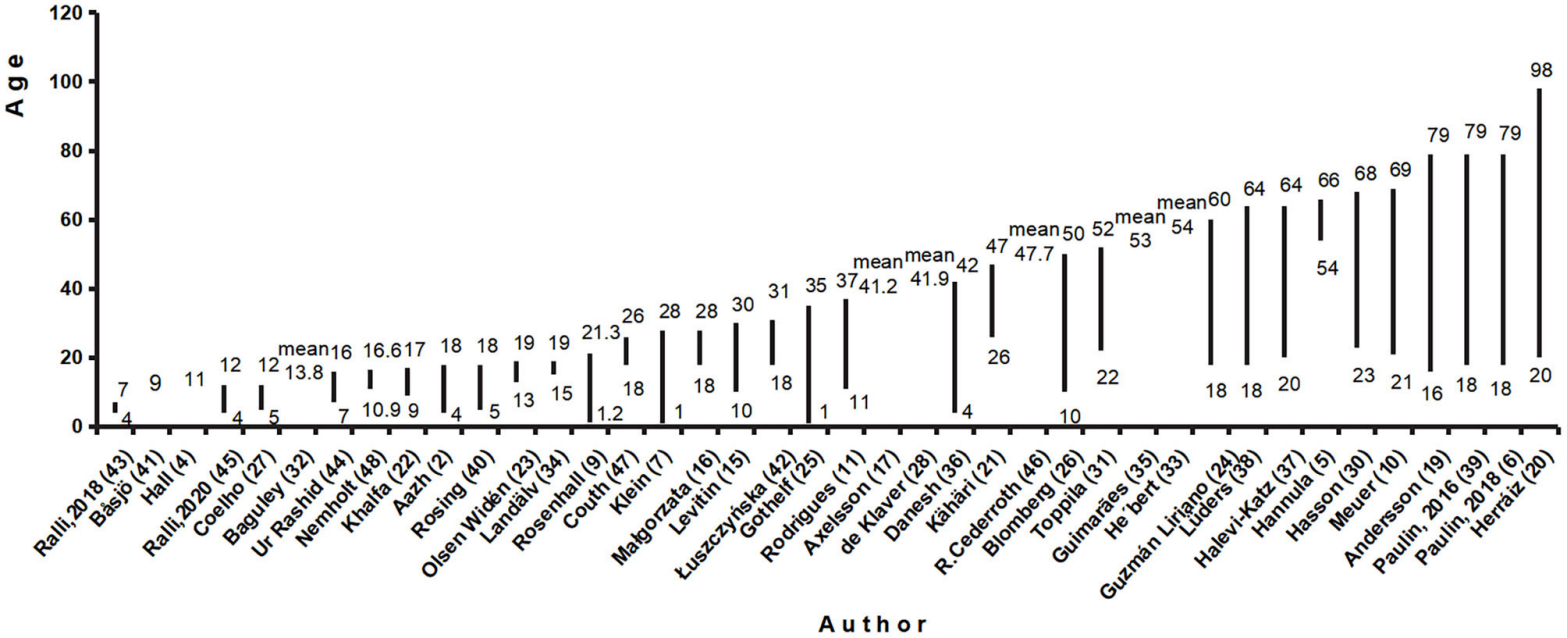
Age/Age range of included studies.

As shown in Figure 3, most of the studies (*n* = 33) (2, 5–7, 9–11, 15, 16, 19–27, 30–32, 34, 36–40, 42–45, 47, 48) focused on a specific age range of people; two studies (4, 41) focused on specific age groups and five studies (17, 28, 33, 35, 46) provided the average age of the subjects, only two studies (18, 29) did not provide age information. These two studies could not be sorted into subgroups to compare by age.

Among these studies, seven pairs of studies had similar average age or age ranges: (9–17, 7–16) (22, 44) (4–18, 5–18) [2, (40)], (13–19, 15–19) (23, 34) (41.2, 41.9) [17, (28)], (53, 54) (33, 35), (20–64,18–64, 18–60) (24, 37, 38), and (21–69, 23–68) (10, 30). The prevalence rates in the first group were 63% (28) and 35% (36). The prevalence rates in the second group were 58% (2) and 12.8% (33). The prevalence rates in the third group were 17.1% (42) and 3.3% (44). The prevalence rates in the fourth group were 3.8% (17) and 38% (39). The prevalence rates in the fifth group were 60% (34) and 18.4% (32). The prevalence rates in the sixth group were 40.0% (22), 67.0% (23), 5.5% (37). The prevalence rates in the seventh group were 24.9% (10) and 14% (30).

Three studies were focused on 17 ± 1 to 79-year-old people. The respective prevalence in these studies was 8.6% (19), 11.1% (39), and 5.5% (6). Two of these studies (6, 39), which were conducted within a 2-year interval, used the same questionnaire. Hyperacusis patients in two studies all gave the affirmative response to the question: “Do you have a hard time tolerating everyday sounds that you believe most other people can tolerate?” and the prevalence in these studies was 11.1% (39) and 5.5% (6) respectively. In another study, the prevalence of hyperacusis was determined by a response of “yes” to the question regarding sensitivity to any sounds, which yielded a prevalence rate of 8.6% (19).

Subjects were divided into Internet groups and a mail group in Andersson's et al. study (19), which were divided into three groups according to age (16–30, 31–50, 51–79), and the prevalence in these three groups was found to be 6.0%, 11.0%, and 15.0%.

#### Gender

Only six (4–6, 21, 26, 35) provided a comparison of male and female prevalence, Kahari's et al. (21), Blomberg's et al. (26), and Paulin's et al. (6) studies suggest that the prevalence in females is higher than in males. Additionally, only one study's subjects are 11-year-old children (4). This study demonstrated higher prevalence in men than in women. Two other studies (5, 35) provided a concrete prevalence of hyperacusis in men and women, with Hannula et al. (5) finding that male prevalence was 11.5% and the prevalence in women was 21.8% (female prevalence was greater than male prevalence), while contrastingly, Guimaraes et al. (35) found that male prevalence was 22.4%, while the prevalence of women was 15.3% (male prevalence was greater than prevalence in women). Although these two studies provided specific instances across gender demographics, Guimaraes' et al. study (35) was conducted on tinnitus patients, while Hannula's et al. study (5) was conducted on older people and thus had less comparative value.

#### Hearing

Studies have also been conducted on only the HD population [self-reported hearing disorders (10) and functional hearing loss (FHL) (44)], all of which have a high prevalence of hyperacusis. The correlation between the prevalence of hyperacusis and HD in other three studies was summarized by two reviewers (17, 20, 27), which found that the prevalence of hyperacusis combined with hearing loss in musicians is 11.3% (17), the prevalence of hyperacusis in patients with hearing loss in children is 6.6% (27), and in a study regarding prevalence of hyperacusis in patients with tinnitus, the prevalence of hyperacusis is 63% (20) and the prevalence of hearing loss is as high as 83% (20). Andersson et al. (19) also studied the prevalence in the mixed population. The subjects included people with normal hearing and people with hearing loss. This study found that the prevalence in people with normal hearing was 6.8% (19), compared to 19.4% (19) in people with hearing loss.

#### People With Special Diseases

Four studies (7, 15, 25, 26) have examined the prevalence of hyperacusis in patients with WS, with results ranging from 4.7% (15) to 95% (7). Seven studies (2, 20, 32, 33, 35, 40, 46) conducted hyperacusis research on patients with tinnitus, with the prevalence ranging from 12.8% (40) to 63% (20), and Aazh et al. (2) found that 17% of patients with tinnitus had severe hyperacusis. Guimaraes et al. also classified the prevalence of hyperacusis in tinnitus patients by gender, finding a rate of 22.4% in men with tinnitus and 15.3% in women (35). In the study conducted by Rosing, 15.7% of subjects showed tinnitus and hyperacusis at the same time (40). What Cederroth's et al. study demonstrated was that the prevalence of hyperacusis increased with the severity of tinnitus and was much higher in tinnitus patients than in the non-tinnitus population (46). Rosenhall et al. (9) and Khalfa et al. (22) studied the prevalence of hyperacusis in children with autism, in which Rosenhall et al. (9) included 199 subjects with a prevalence of 18.0% compared with 0% in the control group, while Khalfa et al. (22) only included 20 subjects with the prevalence of 63.0% compared with 27.0% in the control group. Herraiz et al. (20) found in his study that tinnitus patients not only have a 63% prevalence of hyperacusis but also an 83% prevalence of hearing loss. Additionally, the prevalence of hyperacusis in patients with CRPS related dystonia, Bell's palsy, AS, self-reported hearing disorders, ADHD, and FHL ranged from 5.5% (24) to 69% (36). However, as these studies are about separate conditions, no comparison is proposed.

#### Special Occupational Group

In studying prevalence of hyperacusis, a number of researchers focused on the occupations regularly exposed to sound. The studies (11, 17, 21, 29–31, 37, 38, 42, 47) conducted on musicians and music students, with the prevalence rating from 3.8% (17) to 67% (38), and another study's prevalence of teachers was 24.9% (10). In accordance with a guideline from Kähärit, a professional pop/rock/jazz musician was considered, Halevi-Katz et al. (37) find the prevalence among drummers was as high as 40%. Without using a strict definition of musician, Luders found that musicians had a prevalence of 67%, with the highest rates in people who play amplified instruments (38). In both controlled studies of music students (42) and musicians (47), rates were higher than in the control group. Meuer conducted a survey on the hyperacusis prevalence among teachers (10). He found that although not significant on a very low level, age, period of occupation, and the daily working hours correlate positively with the Mini-TQ data (Mini-Tinnitus Questionnaire), and in the Mini-TQ, groups including hyperacusis scored considerably higher than those excluding hyperacusis.

### Missing Data

There were three conference articles that we did not search for. We attempted to contact the authors, but received no response.

In the results description of two studies, (19, 27) the number of subjects is not consistent with that mentioned in the method. And in another study, (30) the genders of two subjects are unclear. We contacted the authors about this missing data with no results.

Although the website of the questionnaire was given in a study (29), we could not find it according to the website, and there was no detailed content of the questionnaire in the text. In this regard, we contacted the author, but did not get a reply.

## Discussion

Our extensive retrieval of hyperacusis literature finds no existing scoping review of its prevalence. This review, which evaluated 42 studies, is the first scoping review of hyperacusis prevalence in both special and general populations. The prevalence of hyperacusis was discussed in groups and even subgroups, which greatly increased the credibility of our review.

### Characteristics of Prevalence

It was found that the prevalence of hyperacusis is related to age, gender, hearing, comorbidities, and occupation. Overall, the prevalence increases with age, which may be due to the declining function of the medial olivocochlear efferent system with age (3). While in the general population, adolescents also have a higher prevalence. In the population with hearing disorders, teachers have a higher prevalence of hyperacusis. Among all people with concomitant diseases, young patients with WS had the highest prevalence of hyperacusis. Musicians between the ages of 18 and 64 had the highest prevalence. Among people with specific occupations, as working hours increase, the time spent receiving sound or noise increases and the intensity of sound increases as well, the prevalence of tinnitus, hyperacusis and other related symptoms is higher.

**Figure 4:**
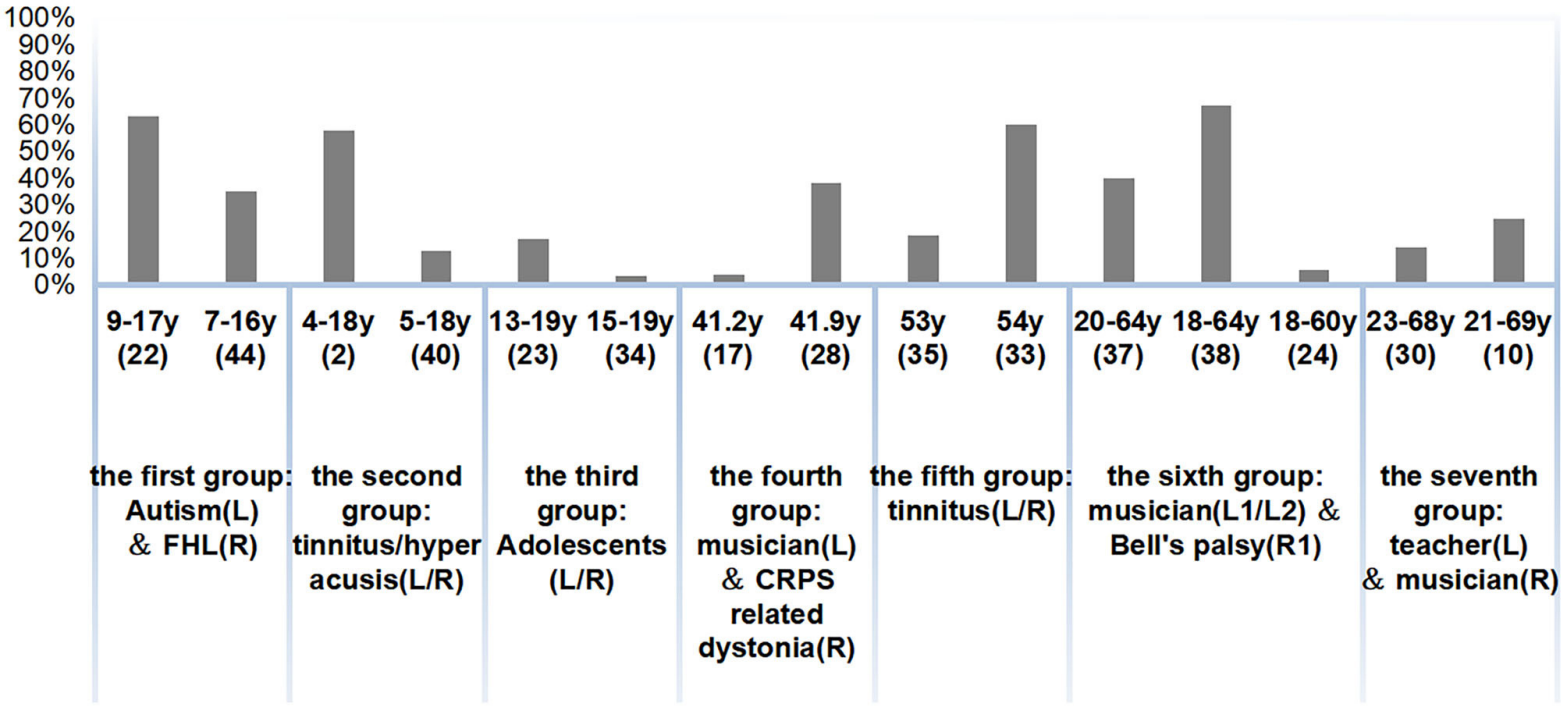
Prevalence of hyperacusis in different age/age range. R, Right; L, Left.

The comparison of prevalence in seven similar age groups is mentioned above (Figure 4). There were three groups of patients of similar ages with comorbid diseases that were partially or completely the same (the second, third, and fifth), but the difference in prevalence is large and difficult to compare. The first group shows that children with autism had a higher prevalence than children with FHL. The fourth group of studies demonstrated that patients with CRPS related-dystonia had a higher prevalence than musicians, a group whose prevalence was relatively high. In the sixth group, however, the musicians had a much higher prevalence of hyperacusis than the Bell's palsy patients. As can be seen from the seventh group of studies, teachers had a higher prevalence than musicians when comparing the prevalence of disease among special occupational groups.

Several studies have shown that women have a higher prevalence than men (5, 6, 21, 26). Among WS patients, the prevalence in female patients was higher than in male patients, and adult female patients have a higher prevalence than young patients and adult male patients (26). Only two studies, with patients of ages 11 and 53, found a higher prevalence in men than in women (4, 35). Although the selected studies did not include people under the age of 10 or over the age of 79, female patients are already clearly at a higher risk of hyperacusis.

Subjects in this review included people with normal hearing, people with disordered hearing, and a mixture of both. Although sensorineural hearing loss may be accompanied by “loudness recruitment,” loudness “catches up” and the person with hearing impairment perceives high-level sounds with a loudness approaching that for listeners with normal hearing, which called “complete” recruitment, and no hyperacusis is present (3, 33). In contrast, the prevalence of hyperacusis was still higher in people with hearing disorders than in people with normal hearing. This is consistent with Andersson's et al. findings, in which the prevalence was 6.8% in the normal hearing population and 19.4% in hearing loss population (19). Hyperacusis, in turn, is considered as a precursor to tinnitus (49), and this conclusion to some extent is consistent with Rosing's finding that tinnitus has the highest prevalence among hearing impaired children (12).

In the population with special diseases [WS (7, 15, 25, 26), tinnitus (2, 20, 32, 33, 35, 40, 46), and autism (9, 22)], the prevalence is mostly higher than in the general population. It is found that tinnitus patients are prone to hyperacusis, and there is a high probability of severe hyperacusis, which is consistent with the hypothesis that tinnitus and hyperacusis may both result from an increase of central gain (33). In randomized controlled trials of WS vs. the general population (7) and randomized controlled trials of autism vs. the general population (9), the hyperacusis prevalence in WS and autism patients is higher than in the general population. These diseases have become risk factors for hyperacusis. Although some studies do not set a control group, it can be seen from the different studies of different populations in this review that the prevalence of hyperacusis in other patients is also significantly higher than that in the general population, such as patients with ADHD, AS, and CRPS related dystonia.

For musicians, music students, and teachers who have been exposed to noise for a long time, the prevalence of the disease is higher than the general population. People working in these professions will not only receive daily noise in the general population (vehicle noise, the noise of construction equipment, and household appliances noise, etc), but also in their occupational environments. They will thus be exposed to noise of wider frequency, different volumes, and longer duration. They are exposed to a great deal of sound during the course of their employment, which explains the increase in prevalence.

### Limitations of This Study

First of all, the research purposes of all the studies are different, which makes it difficult to extract data. Although the two authors have a good consensus, sometimes there are disagreements. For the included data with inconsistent opinions, a temporary reviewer is needed to judge.

Secondly, it can be found from the area in which the studies is distributed (Figure 2) that most of the literature come from western countries, while few studies from other regions are included.

Finally, this review restricted the included literature to be in English, so the included articles did not include non-English literature, which may lead to an incomplete summary of current hyperacusis findings.

### Future Perspectives and Conclusions

The diverse definition and diagnosis way between the studies cannot be ignored, which may lead to differences in prevalence. It is not difficult to find from the included studies that not every study has a clear definition of hyperacusis, and there is no universal definition at present. Taken together with the definitions already mentioned in the studies included in this review, excessive sensitivity to sound that is acceptable to the general population should be a generally acceptable definition.

What is undoubtedly the most intuitionistic for the measurement and diagnosis of hyperacusis is the audiology examination. In particular, pure tone audiometry can give the study object different loudness and frequency of the stimulus sound, which can measure the severity of hyperacusis. According to the included studies, it is an effective method to measure and diagnose hyperacusis by audiology examination method combined with hyperacusis related questionnaire, such as Hyperacusis Questionnaire (50), which was further specified in Fackrell's review as LDL and self-report questionnaire (51). On the one hand, the research object can be assessed in a more comprehensive way; on the other hand, the questionnaire survey can reflect whether the audiology examination results are consistent with the subjective symptoms of the research object. And for subjects with low cognitive ability, their closest contacts should participate in the whole study process as far as possible to ensure the maximum accuracy of the results. At present, these are still in the groping stage. In the future, more scholars need to further explore and improve it bit by bit.

According to the current scoping review, the prevalence of hyperacusis in different groups have been studied and it is found that the prevalence varies greatly among these groups. The prevalence of hyperacusis is related to several factors (special occupation, special diseases, gender, age, hearing, etc). But the most important next step is to develop unified standards for the definition and diagnosis of hyperacusis. This will improve the comparability of each study, so that reviews of hyperacusis prevalence can be more accurate and more credible.

## Data Availability Statement

The original contributions presented in the study are included in the article/supplementary material, further inquiries can be directed to the corresponding author/s.

## Author Contributions

DL, LL, and JMP had the idea for the article. JR, TXu, TXi, and YX performed the literature search and data extraction. JR and TXu drafted the work. DL critically revised the work. All authors contributed to the study conception, design, read, and approved the final manuscript.

## Conflict of Interest

The authors declare that the research was conducted in the absence of any commercial or financial relationships that could be construed as a potential conflict of interest.

## Publisher's Note

All claims expressed in this article are solely those of the authors and do not necessarily represent those of their affiliated organizations, or those of the publisher, the editors and the reviewers. Any product that may be evaluated in this article, or claim that may be made by its manufacturer, is not guaranteed or endorsed by the publisher.

## Abbreviations

WS: Williams syndrome
ADHD: attention deficit hyperactivity disorder
CRPS related dystonia: complex regional pain syndrome related dystonia
AS: Asperger's syndrome
LDL: loudness discomfort level
HD: hearing disorders
UCL: uncomfortable loudness
ULL: uncomfortable loudness level
FHL: functional hearing loss.

## References

[1] Di Stadio A Dipietro L Ricci G Della Volpe A Minni A Greco A. Hearing loss, tinnitus, hyperacusis, and diplacusis in professional musicians: a systematic review. Int J Environ Res Public Health. (2018) 15:2120. 10.3390/ijerph15102120 30261653 PMC6209930

[2] Aazh H McFerran D Moore BCJ. Uncomfortable loudness levels among children and adolescents seeking help for tinnitus and/or hyperacusis. Int J Audiol. (2018) 57:618–23. 10.1080/14992027.2018.1453617 29688102

[3] Tyler RS Pienkowski M Roncancio ER Jun HJ Brozoski T Dauman N. A review of hyperacusis and future directions: part I. Definitions and manifestations. Am J Audiol. (2014) 23:402–19. 10.1044/2014_AJA-14-0010 25104073

[4] Hall AJ Humphriss R Baguley DM Parker M Steer CD. Prevalence and risk factors for reduced sound tolerance (hyperacusis) in children. Int J Audiol. (2016) 55:135–41. 10.3109/14992027.2015.1092055 26642866

[5] Hannula S Bloigu R Majamaa K Sorri M Maki-Torkko E. Self-reported hearing problems among older adults: prevalence and comparison to measured hearing impairment. J Am Acad Audiol. (2011) 22:550–9. 10.3766/jaaa.22.8.7 22031679

[6] Paulin J Nordin M Nyback MH Nordin S. Associations between hyperacusis and psychosocial work factors in the general population. Int Arch Occup Environ Health. (2018) 92:59–65. 10.1007/s00420-018-1356-x 30194539 PMC6323093

[7] Klein AJ Armstrong BL Greer MK Brown FR III. Hyperacusis and otitis media in individuals with Williams syndrome. J Speech Hear Disord. (1990) 55:339–44. 10.1044/jshd.5502.339 2329796

[8] Fackrell K Stratmann L Gronlund TA Hoare DJ Hyperacusis Priority Setting Partnership Steering G. Top ten hyperacusis research priorities in the UK. Lancet. (2019) 393:404–5. 10.1016/S0140-6736(18)32616-3 30722956

[9] Rosenhall U Nordin V Sandstrom M Ahlsen G Gillberg C. Autism and hearing loss. J Autism Dev Disord. (1999) 29:349–57. 10.1023/a:1023022709710 10587881

[10] Meuer SP Hiller W. The impact of hyperacusis and hearing loss on tinnitus perception in German teachers. Noise Health. (2015) 17:182–90. 10.4103/1463-1741.160682 26168948 PMC4900479

[11] Rodrigues MA Amorim M Silva MV Neves P Sousa A Inacio O. Sound levels and risk perceptions of music students during classes. J Toxicol Environ Health A. (2015) 78:825–39. 10.1080/15287394.2015.1051174 26167749

[12] Rosing SN Schmidt JH Wedderkopp N Baguley DM. Prevalence of tinnitus and hyperacusis in children and adolescents: a systematic review. BMJ Open. (2016) 6:e010596. 10.1136/bmjopen-2015-010596 27259524 PMC4893873

[13] Baguley DM Hoare DJ. Hyperacusis: major research questions. HNO. (2018) 66:358–63. 10.1007/s00106-017-0464-3 29392341 PMC5928178

[14] Arksey H O'Malley L. Scoping studies: towards a methodological framework. Int J Soc Res Methodol. (2005) 8:19–32. 10.1080/1364557032000119616

[15] Levitin DJ Cole K Lincoln A Bellugi U. Aversion, awareness, and attraction: investigating claims of hyperacusis in the williams syndrome phenotype. J Child Psychol Psychiatry. (2005) 46:514–23. 10.1111/j.1469-7610.2004.00376.x 15845131

[16] Pawlaczyk-Luszczyska M Zaborowski K Zamojska-Daniszewska M Rutkowska-Kaczmarek P Dudarewicz A Sliwinska-Kowalska M. Hearing status in young people using portable audio players. Arch Acoust. (2017) 42:113–20. 10.1515/aoa-2017-0012

[17] Axelsson A Eliasson A Israelsson B. Hearing in pop/rock musicians: a follow-up study. Ear Hear. (1995) 16:245–53. 10.1097/00003446-199506000-00001 7672473

[18] Skarzynski H Rogowski M Bartnik G Fabijanska A. Organization of tinnitus management in Poland. Acta Otolaryngol. (2000) 120:225–6. 10.1080/000164800750000973 11603778

[19] Andersson G Lindvall N Hursti T Carlbring P. Hypersensitivity to sound (hyperacusis): a prevalence study conducted via the internet and post. Int J Audiol. (2002) 41:545–54. 10.3109/14992020209056075 12477175

[20] Herraiz C Hernandez Calvin J Plaza G Toledano A de los Santos G. [Study of hyperacusis at a tinnitus unit]. Acta Otorrinolaringol Esp. (2003) 54:617–22. 10.1016/s0001-6519(03)78458-1 14992115

[21] Kaharit K Zachau G Eklof M Sandsjo L Moller C. Assessment of hearing and hearing disorders in rock/jazz musicians. Int J Audiol. (2003) 42:279–88. 10.3109/14992020309078347 12916701

[22] Khalfa S Bruneau N Roge B Georgieff N Veuillet E Adrien JL. Increased perception of loudness in autism. Hear Res. (2004) 198:87–92. 10.1016/j.heares.2004.07.006 15617227

[23] Widen SE Erlandsson SI. Self-reported tinnitus and noise sensitivity among adolescents in Sweden. Noise Health. (2004) 7:29–40. 15703147

[24] Liriano RYG Magalhães SLBd Barros F Testa JRG Fukuda Y. Relação da presença de hiperacusia em pacientes com paralisia facial periférica de bell. Rev Bras Otorrinolaringol. (2004) 70:776–9. 10.1590/s0034-72992004000600012

[25] Gothelf D Farber N Raveh E Apter A Attias J. Hyperacusis in williams syndrome: characteristics and associated neuroaudiologic abnormalities. Neurology. (2006) 66:390–5. 10.1212/01.wnl.0000196643.35395.5f 16476938

[26] Blomberg S Rosander M Andersson G. Fears, hyperacusis and musicality in williams syndrome. Res Dev Disabil. (2006) 27:668–80. 10.1016/j.ridd.2005.09.002 16269236

[27] Coelho CB Sanchez TG Tyler RS. Hyperacusis, sound annoyance, and loudness hypersensitivity in children. Prog Brain Res. (2007) 166:169–78. 10.1016/S0079-6123(07)66015-4 17956781

[28] de Klaver MJ van Rijn MA Marinus J Soede W de Laat JA van Hilten JJ. Hyperacusis in patients with complex regional pain syndrome related dystonia. J Neurol Neurosurg Psychiatry. (2007) 78:1310–3. 10.1136/jnnp.2006.111609 17470470 PMC2095603

[29] Laitinen H Poulsen T. Questionnaire investigation of musicians' use of hearing protectors, self reported hearing disorders, and their experience of their working environment. Int J Audiol. (2008) 47:160–8. 10.1080/14992020801886770 18389411

[30] Hasson D Theorell T Liljeholm-Johansson Y Canlon B. Psychosocial and physiological correlates of self-reported hearing problems in male and female musicians in symphony orchestras. Int J Psychophysiol. (2009) 74:93–100. 10.1016/j.ijpsycho.2009.07.009 19666059

[31] Toppila E Koskinen H Pyykko I. Hearing loss among classical-orchestra musicians. Noise Health. (2011) 13:45–50. 10.4103/1463-1741.74001 21173486

[32] Baguley DM Bartnik G Kleinjung T Savastano M Hough EA. Troublesome tinnitus in childhood and adolescence: data from expert centres. Int J Pediatr Otorhinolaryngol. (2013) 77:248–51. 10.1016/j.ijporl.2012.11.009 23245492

[33] Hebert S Fournier P Norena A. The auditory sensitivity is increased in tinnitus ears. J Neurosci. (2013) 33:2356–64. 10.1523/JNEUROSCI.3461-12.2013 23392665 PMC6619157

[34] Landalv D Malmstrom L Widen SE. Adolescents' reported hearing symptoms and attitudes toward loud music. Noise Health. (2013) 15:347–54. 10.4103/1463-1741.116584 23955132

[35] Guimaraes AC Carvalho GM Voltolini MM Zappelini CE Mezzalira R Stoler G. Study of the relationship between the degree of tinnitus annoyance and the presence of hyperacusis. Braz J Otorhinolaryngol. (2014) 80:24–8. 10.5935/1808-8694.20140007 24626888 PMC9443979

[36] Danesh AA Lang D Kaf W Andreassen WD Scott J Eshraghi AA. Tinnitus and hyperacusis in autism spectrum disorders with emphasis on high functioning individuals diagnosed with asperger's syndrome. Int J Pediatr Otorhinolaryngol. (2015) 79:1683–8. 10.1016/j.ijporl.2015.07.024 26243502

[37] Halevi-Katz DN Yaakobi E Putter-Katz H. Exposure to music and noise-induced hearing loss (NIHL) among professional pop/rock/jazz musicians. Noise Health. (2015) 17:158–64. 10.4103/1463-1741.155848 25913555 PMC4918652

[38] Luders D Goncalves CG Lacerda AB Silva LS Marques JM Sperotto VN. Occurrence of tinnitus and other auditory symptoms among musicians playing different instruments. Int Tinnitus J. (2016) 20:48–53. 10.5935/0946-5448.20160009 27488994

[39] Paulin J Andersson L Nordin S. Characteristics of hyperacusis in the general population. Noise Health. (2016) 18:178–84. 10.4103/1463-1741.189244 27569405 PMC5187659

[40] Rosing SN Kapandais A Schmidt JH Baguley DM. Demographic data, referral patterns and interventions used for children and adolescents with tinnitus and hyperacusis in Denmark. Int J Pediatr Otorhinolaryngol. (2016) 89:112–20. 10.1016/j.ijporl.2016.07.036 27619040

[41] Basjo S Moller C Widen S Jutengren G Kahari K. Hearing thresholds, tinnitus, and headphone listening habits in nine-year-old children. Int J Audiol. (2016) 55:587–96. 10.1080/14992027.2016.1190871 27329351 PMC4989862

[42] Pawlaczyk-Luszczynska M Zamojska-Daniszewska M Dudarewicz A Zaborowski K. Exposure to excessive sounds and hearing status in academic classical music students. Int J Occup Med Environ Health. (2017) 30:55–75. 10.13075/ijomeh.1896.00709 28220907

[43] Ralli M Greco A Altissimi G Tagliaferri N Carchiolo L Turchetta R. Hyperacusis in children: a preliminary study on the effects of hypersensitivity to sound on speech and language. Int Tinnitus J. (2018) 22:10–8. 10.5935/0946-5448.20180002 29993211

[44] Rashid SMU Mukherjee D Ahmmed AU. Auditory processing and neuropsychological profiles of children with functional hearing loss. Int J Pediatr Otorhinolaryngol. (2018) 114:51–60. 10.1016/j.ijporl.2018.07.054 30262367

[45] Ralli M Romani M Zodda A Russo FY Altissimi G Orlando MP. Hyperacusis in children with attention deficit hyperactivity disorder: a preliminary study. Int J Environ Res Public Health. (2020) 17:3045. 10.3390/ijerph17093045 32349379 PMC7246428

[46] Cederroth CR Lugo A Edvall NK Lazar A Lopez-Escamez J-A Bulla J. Association between hyperacusis and tinnitus. J Clin Med. (2020) 9:2412. 10.3390/jcm9082412 32731492 PMC7465629

[47] Couth S Prendergast G Guest H Munro KJ Moore DR Plack CJ. Investigating the effects of noise exposure on self-report, behavioral and electrophysiological indices of hearing damage in musicians with normal audiometric thresholds. Hear Res. (2020) 395:108021. 10.1016/j.heares.2020.108021 32631495

[48] Nemholt S Schmidt JH Wedderkopp N Baguley DM. A cross-sectional study of the prevalence and factors associated with tinnitus and/or hyperacusis in children. Ear Hear. (2020) 41:344–55. 10.1097/AUD.0000000000000759 31365354 PMC7664713

[49] Jastreboff PJ Hazell JW. A neurophysiological approach to tinnitus: clinical implications. Br J Audiol. (1993) 27:7–17. 10.3109/03005369309077884 8339063

[50] Khalfa S Dubal S Veuillet E Perez-Diaz F Jouvent R Collet L. Psychometric normalization of a hyperacusis questionnaire. ORL J Otorhinolaryngol Relat Spec. (2002) 64:436–42. 10.1159/000067570 12499770

[51] Fackrell K Potgieter I Shekhawat GS Baguley DM Sereda M Hoare DJ. Clinical interventions for hyperacusis in adults: a scoping review to assess the current position and determine priorities for research. Biomed Res Int. (2017) 2017:2723715. 10.1155/2017/2723715 29312994 PMC5654244

